# A Patient With Fever, Loose Motions and Jaundice: Hickam’s Dictum or Occam's Razor

**DOI:** 10.7759/cureus.23295

**Published:** 2022-03-18

**Authors:** Mohammad Noor, Fawad Rahim, Said Amin, Raza Ullah, Sheraz Zafar

**Affiliations:** 1 Internal Medicine, Khyber Girls Medical College, Peshawar, PAK; 2 Internal Medicine, Hayatabad Medical Complex, Peshawar, PAK; 3 Critical Care Medicine, Hayatabad Medical Complex, Peshawar, PAK; 4 Critical Care Medicine, Khyber Girls Medical College, Peshawar, PAK

**Keywords:** drug susceptibility testing and antibiotic resistance, coexistent disease, xdr enteric fever, leptospira, salmonella enterica serovar typhi

## Abstract

Infectious diseases like malaria, typhoid, leptospirosis, and dengue fever are the leading causes of morbidity and mortality in developing countries like Pakistan. Although rare, it is possible to have coinfection with organisms that are endemic in a region, causing diagnostic and therapeutic dilemmas.

Leptospirosis is caused by Gram-negative spirochetes. Leptospira are widely distributed and are transmitted by contamination of water and food by the urine of infected animals like rodents. Leptospirosis is characterized by fever, body aches, abdominal pain, and hepatic and renal involvement. Laboratory abnormalities include cytopenia, elevated bilirubin, alanine aminotransferase, and abnormal renal function tests.

Typhoid fever is caused by Salmonella typhi (S. typhi), which is transmitted by fecal contamination of drinking water and food items. The clinical manifestations of typhoid fever include fever, abdominal pain, and diarrhea. Laboratory abnormalities include cytopenia and mildly deranged liver function tests. A strain of S. typhi resistant to all antibiotics except azithromycin and carbapenems was isolated in 2016 in Pakistan.

Most of the clinical manifestations and laboratory abnormalities of leptospirosis and typhoid fever overlap. There have been case reports of coinfection of S. typhi and Leptospira, but there is no report of coinfection of extensively drug-resistant (XDR) S. typhi and Leptospira. We present a case of a 20-year-old man with fever, loose motions, and jaundice from Peshawar, Pakistan who had coinfection of Leptospira and XDR S. typhi. The attending physicians should adopt Hickam’s dictum instead of Occam's razor approach.

## Introduction

Infectious diseases are the leading cause of morbidity and mortality in developing countries. Malaria, typhoid, leptospirosis, and dengue fever are the most frequent infectious diseases in Pakistan [1]. Coinfections cause diagnostic and therapeutic challenges. These can also have an unfavorable impact on the patient compared to a single infection [2].

Leptospirosis is caused by Gram-negative, motile, spiral shape spirochetes. Leptospira are widely distributed in nature and are more frequently reported from both rural and urban areas of tropical and subtropical regions. Rodents, farm animals, and dogs can get infected by the spirochete. Leptospira colonize the urinary tract of these animals and are passed in the urine [3]. Leptospirosis is characterized by fever, chills, headaches, body aches, anorexia, variable degree of abdominal pain, and hepatic and renal involvement. Investigations reveal a variable degree of cytopenia, abnormal liver and renal functions [4].

Typhoid fever has been a major public health problem in the past globally. It is still a major public health issue in developing countries with 21.6 million cases and 250,000 deaths every year [5]. In February 2018, an outbreak of Salmonella typhi (S. typhi) which was resistant to chloramphenicol, ampicillin, trimethoprim-sulfamethoxazole, fluoroquinolones, and third-generation cephalosporins was reported in Pakistan [6]. The National Institute of Health (NIH), Islamabad reported a total of 14,360 extensively drug-resistant (XDR) S. typhi in Karachi till June 2021 [7]. Travelers, particularly those planning family visits to Pakistan, have been warned by the Center for Disease Control about the risk of XDR S. typhi. Cases of XDR S. typhi have been reported among travelers from Pakistan to the United States of America (USA), the United Kingdom, and Canada [8]. The clinical manifestations of S. typhi infection are fever, anorexia, body aches, abdominal pain, and diarrhea. Occasionally some patients develop gastrointestinal bleeding, ileal perforation, peritonitis and encephalopathy. Laboratory abnormalities include leukopenia and raised alanine aminotransferase levels.

Leptospirosis and typhoid fever are caused by exposure to contaminated water and food items. The urine of animals infected by Leptospira can contaminate water, which is the source of infection in occupational workers. Occupational groups such as farmers, veterinarians, animal shelter employees, hunters, and abattoir workers are at substantial risk of infection. Nonoccupational outbreaks occur after flooding during the rainy season leading to contamination of food items [9]. Similarly, S. typhi is transmitted by fecal contamination of drinking water and food items. This is extremely common in densely populated slums in big cities of developing countries like Pakistan.

It is possible to be coinfected with organisms that are endemic in a region. The clinical manifestations and laboratory abnormalities of many infectious diseases are identical. The attending physician should adopt Hickam’s dictum (several reasons for multiple symptoms) instead of Occam's razor (single reason for multiple symptoms). We present a case of a 20-year-old man with fever, loose motions, and jaundice from Peshawar, Pakistan who had coinfection of Leptospira and XDR S. typhi. To date, there is no reported case of coinfection of XDR S. typhi and Leptospira.

## Case presentation

A 20-year-old man was admitted with complaints of fever for 10 days, and loose stools and jaundice for seven days. He was in his usual state of health 12 days before admission when he returned from a congregation in Punjab, Pakistan. Two days after return, he developed a high-grade fever (documented up to 104 F) associated with rigors, chills, and sweating. The fever was not associated with any focal symptoms. He visited a local health facility where he was prescribed oral ciprofloxacin and sulfadoxine/pyrimethamine. Three days into the fever, he developed watery stools, three to four episodes a day, associated with diffuse abdominal pain. He developed jaundice at the same time. A physician reviewed him at the district general hospital and advised him oral cefixime, artemether/lumefantrine, and domperidone. Then he developed headache, vomiting, and dry cough. His medical and surgical history was unremarkable except for an uneventful appendectomy 10 years ago. He was admitted to the district general hospital and received parenteral ceftriaxone and artemether. His investigations at the district general hospital are summarized in Table 1.

**Table 1:** Preliminary Investigations at District General Hospital g/dL: Gram/deciliter, mcL: Microliter, ELISA: Enzyme-linked immunosorbent assay, HBsAg: Hepatitis B surface antigen, HCV: Hepatitis C virus, HIV: Human immunodeficiency virus, IU/L: International unit/liter, NS: Non-structural, IgM: Immunoglobulin M, PA: Posterior-anterior

Investigations	Reference range	Results
Hemoglobin (g/dL)	13.5 – 17.5	11.2
Platelet count (x10^3^/mcL)	150 – 450	37,000
White cell count (x10^3^/mcL)	4.5 – 11	4.1
Neutrophils (%)	40 – 60 %	81%
Lymphocytes (%)	20 – 40 %	16%
Monocytes (%)	2 – 8 %	02 %
Eosinophils (%)	1 – 4 %	01 %
HBsAg (ELISA)	Non-Reactive	Non-Reactive
Anti-HCV (ELISA)	Non-Reactive	Non-Reactive
Anti-HIV (ELISA)	Non-Reactive	Non-Reactive
Total Bilirubin (mg/dL)	0.2 – 1.2	12.6
Alanine aminotransferase (IU/L)	< 45	218
Alkaline phosphatase (IU/L)	30 – 120	360
Dengue NS-1 antigen	Non-Reactive	Non-Reactive
Dengue IgM antibodies	Non-Reactive	Non-Reactive
Urinalysis	Normal	
Malarial Parasite	Not seen	
Ultrasound Abdomen and Pelvis	Mild hepatosplenomegaly	
X-Ray Chest (PA) view	Normal	
Bone marrow biopsy	Hypercellular bone marrow, thrombocytopenia suggestive of peripheral destruction	

With no improvement despite four days stay at the district general hospital, he was shifted to a tertiary care hospital (Hayatabad Medical Complex, Peshawar, Pakistan). On arrival, he was pale, icteric, and had a coated tongue with a temperature of 103 F, a pulse of 80/min, and blood pressure of 110/70 mmHg. His respiratory rate was 15/min, and his oxygen saturation was 98% on room air. He had tender hepatomegaly and appendectomy scar with no evidence of intrabdominal collection. His cardiac, respiratory, and neurological examination was unremarkable.

Blood, urine, and stool cultures were sent. Based on his clinical presentation and common local infectious diseases, viral hepatitis, liver abscess, dengue fever, malaria, infectious mononucleosis, leptospirosis, cholera, typhoid fever, and hemolysis due to glucose-6-phosphate dehydrogenase deficiency were considered in the differential diagnosis. His investigations at the tertiary care hospital are summarized in Table 2.

**Table 2:** Investigations at Tertiary Care Hospital g/dL: Gram/deciliter, mcL: Microliter, U/L: Unit/liter, mg/dL: milligram/deciliter, IU/L: International unit/liter, PCR: Polymerase chain reaction, PT: Prothrombin time, APTT: Activated partial thromboplastin time, G6PD: Glucose-6-phosphate dehydrogenase, SARS-CoV-2: Severe acute respiratory syndrome coronavirus 2, IgM: Immunoglobulin M, IgG: Immunoglobulin G, ELISA: Enzyme-linked immunosorbent assay, NS: Non-structural

Investigations	Reference range	Results
Hemoglobin (g/dL)	13.5 – 17.5	10.6
Platelet count (x10^3^/mcL)	150 – 450	90,000
White cell count (x10^3^/mcL)	4.5 – 11	7.81
Neutrophils (%)	40 – 60 %	77 %
Lymphocytes (%)	20 – 40 %	20 %
Monocytes (%)	2 – 8 %	02 %
Eosinophils (%)	1 – 4 %	01 %
Reticulocytes (%)	0.5 – 2 %	1.1%
Lactate dehydrogenase (U/L)	140 – 280	712
C-Reactive Protein (mg/dL)	< 0.5	7.2
Total Bilirubin (mg/dL)	0.2 – 1.2	13.4
Direct Bilirubin (mg/dL)	< 0.3	9.9
Alanine aminotransferase (IU/L)	< 45	184
Alkaline phosphatase (IU/L)	30 – 120	391
S. Albumin (g/dL)	3.4 – 5.5	2.0
S. creatinine (mg/dL)	0.5 – 1.2	0.8
Urea (mg/dL)	20 – 40	32
PT (seconds)	12	12
APTT (seconds)	32	30
G6PD (units/gram Hb)	5.5 – 20.5	1.5
SARS–CoV–2 PCR	Negative	Negative
Anti-hepatitis ‘A’ IgM and IgG (ELISA)	Non-reactive	Non-reactive
Anti-leptospiral IgM (ELISA)	Non-reactive	Reactive
Anti-leptospiral IgG (ELISA)	Non-reactive	Non-reactive
Dengue NS1-Antigen and Antibodies (ELISA)	Non-reactive	Non-reactive
Anti-Nuclear Antibody	Non-reactive	Non-reactive
Coomb’s test (direct and indirect)	Negative	Negative
Monospot test	Negative	Negative
Special smear	No abnormal cells	
Stool examination	Numerous pus cells	
Echocardiography	Mild pericardial effusion	
Ultrasound Abdomen and pelvis	Hepatomegaly (18 cm)	
CT chest	Normal	
CT abdomen and pelvis	Hepatosplenomegaly; few mesenteric lymph nodes in the right iliac fossa, largest up to 1.4 cm	
Blood Culture	Gram-Negative Rods of S. typhi sensitive to Meropenem, Azithromycin. Resistant to ciprofloxacin, ceftriaxone, cefuroxime, doxycycline.	
Urine culture	No growth	
Stool culture	No growth of any pathogenic organism	

Parenteral meropenem was started empirically keeping in view resistant typhoid fever, and artemether was continued. His Leptospira IgM (enzyme-linked immunosorbent assay [ELISA]) was reported positive on day 13 and the preliminary report of blood culture revealed gram-negative rods on day 14. The most appropriate antibiotics for the coinfection were discussed with an infectious disease specialist and microbiologist. Artemether was stopped and meropenem was continued as decided by the multidisciplinary team. On day 17, the final report of blood culture revealed XDR S. typhi which was only sensitive to azithromycin, meropenem, and imipenem. It was resistant to all other antibiotics. The case was notified to the public health department. He became afebrile on day eight of meropenem. The patient received 14 days of inpatient care and remained afebrile till discharge. His blood counts and liver functions showed progressive improvement (Table 3). He was discharged and booked for an outpatient visit and vaccination against typhoid fever in four weeks. He was advised to report early should he have any relapse of symptoms. He was educated regarding glucose-6-phosphate-dehydrogenase deficiency and a list of drugs to be avoided was handed over to him. 

**Table 3:** Laboratory Investigations During Hospital Stay g/dL: Gram/deciliter, mcL: Microliter, mg/dL: milligram/deciliter, IU/L: International unit/liter

	Reference Range	Day 3	Day 5	Day 7	Day 9	Day 11	Day 13	Day 14
Hemoglobin (g/dL)	13.5 – 17.5	9.6	10.6	9.4	8.7	9.2	10.1	10.7
Platelet count (x10^3^/mcL)	150 – 450	231	453	341	348	468	432	469
White cell count (x10^3^/mcL)	4.5 – 11	6.5	9.4	8.9	8.6	8.5	8.8	8.7
Total Bilirubin (mg/dL)	0.2 – 1.2	12.2	6.8	5.7	3.3	2.4	2.0	1.6
Alanine aminotransferase (IU/L)	< 45	97	130	108	67	58	49	44
Alkaline phosphatase (IU/L)	30 – 120	342	606	449	348	298	263	211

## Discussion

Since its isolation in 2016 in Pakistan, XDR S. typhi has become a substantial threat to regional and global public health. Apart from typhoid fever, leptospirosis is also endemic in Pakistan. Sometimes, the two infections coexist, making it a diagnostic and therapeutic dilemma that can potentially lead to disability and death.

Coinfection of S. typhi and Leptospira have been reported from the USA, Denmark, Egypt, Tanzania, India, and Korea [10-16]. Salmonella osteomyelitis has been reported in association with leptospirosis in a 16-year-old boy from the USA. The diagnosis was made by culturing S. typhi in the aspirate from an intervertebral disc space abscess. The S. typhi was sensitive to penicillin and quinolones [10]. Rönsholt et al. have reported S. typhi and Leptospira coinfection in a 56-year-old man admitted with septicemia after occupational exposure. Salmonella typhi was cultured from the stool, and Leptospira was demonstrated by polymerase chain reaction. It is worth mentioning that in the case report the authors did not share the sensitivity report of S. typhi [12]. Parker et al. from Egypt have reported only one confirmed case of coinfection of S. typhi and Leptospira based on positive cultures for both organisms out of 1510 patients with acute febrile illness [13]. Biggs et al. from Tanzania reported that three out of 40 confirmed cases of leptospirosis had evidence of coinfection with S. typhi. Since the diagnosis of S. typhi infection was based on serological methods, the authors were unable to establish whether the apparent coinfections represent true simultaneous infections or cross-reactivity between the assays used [14]. Sahu et al. from India have reported a case of S. typhi causing splenic abscess in a patient with leptospirosis. However, the S. typhi was sensitive to ceftriaxone [11]. A case of subacute intestinal obstruction with colitis was reported by Negi et al. from India. It is worthwhile pointing out that the diagnosis of typhoid fever was based on serological tests while blood culture did not grow any organism [15]. Song et al. from Korea reported a 37-year-old man with blood culture positive for S. typhi and positive Leptospira serology. The authors did not share the sensitivity report of S. typhi. The case report dates back to 2010 when XDR S. typhi was not yet reported [16].

We report a case of XDR S. typhi coinfection with Leptospira which has not been reported in the literature so far. The XDR S. typhi was sensitive to azithromycin, meropenem and imipenem. These antibiotics are very expensive and, except azithromycin, must be administered parenterally. Instead of outpatient treatment, the patient would need hospitalization which will overstretch the scarce health resources amidst the pandemic of COVID-19.

Antibiotics including azithromycin and carbapenems have been misused in the COVID-19 pandemic, both in developed and underdeveloped countries. It has been reported from the USA that 90% of patients of COVID-19 received antibiotics while only 7% needed it [17]. The irrational use of antibiotics in the current pandemic is very alarming in Pakistan. It has been reported that Pakistan is the third highest consumer of antibiotics in the world after India and China [18]. It has been reported that 100% of patients of COVID-19 were given antibiotics, especially azithromycin before hospitalization [19]. A case of an azithromycin-resistant strain of XDR S. typhi has already been reported [20]. Resistance to carbapenems has already been observed in nontyphoidal Salmonella serovars, leading to the fear that S. typhi will become resistant to all available antibiotics. There is an urgent need for effective, collective and comprehensive action at both the national and international levels to stop the volcano from erupting. If concrete steps are not taken at this moment, we may return to the pre-antibiotic era as far as S. typhi is concerned. No one is safe till everyone is safe in the global village.

## Conclusions

Despite the high prevalence of many infectious diseases in developing countries, coinfections are reported less frequently. There are case reports of coinfection of non-XDR S. typhi with Leptospira. Since its isolation in 2016, XDR S. typhi coinfection with Leptospira has not been reported. The clinical and laboratory findings of most of the endemic infectious diseases overlap. This may mislead the physicians to overlook the diagnosis of coinfections. Adopting Hickam’s dictum approach rather than Occam's razor will be more appropriate to avoid missing the diagnosis of coinfection.
